# Hollow-Core Photonic Crystal Fiber Gas Sensing

**DOI:** 10.3390/s20102996

**Published:** 2020-05-25

**Authors:** Ruowei Yu, Yuxing Chen, Lingling Shui, Limin Xiao

**Affiliations:** 1 Advanced Fiber Devices and Systems Group, Key Laboratory of Micro and Nano Photonic Structures (MoE), Department of Optical Science and Engineering, Fudan University, Shanghai 200433, China; 17210720016@fudan.edu.cn (R.Y.); 15307110401@fudan.edu.cn (Y.C.); 2 Guangdong Provincial Key Laboratory of Optical Information Materials and Technology, South China Normal University, Guangzhou 510631, China; Shuill@m.scnu.edu.cn; 3 Key Laboratory for Information Science of Electromagnetic Waves (MoE), Fudan University, Shanghai 200433, China; 4 Shanghai Engineering Research Center of Ultra-Precision Optical Manufacturing, Fudan University, Shanghai 200433, China

**Keywords:** hollow-core photonic crystal fibers, optical fiber devices, fiber gas sensing

## Abstract

Fiber gas sensing techniques have been applied for a wide range of industrial applications. In this paper, the basic fiber gas sensing principles and the development of different fibers have been introduced. In various specialty fibers, hollow-core photonic crystal fibers (HC-PCFs) can overcome the fundamental limits of solid fibers and have attracted intense interest recently. Here, we focus on the review of HC-PCF gas sensing, including the light-guiding mechanisms of HC-PCFs, various sensing configurations, microfabrication approaches, and recent research advances including the mid-infrared gas sensors via hollow core anti-resonant fibers. This review gives a detailed and deep understanding of HC-PCF gas sensors and will promote more practical applications of HC-PCFs in the near future.

## 1. Introduction

Gas sensing has a significant impact on a wide range of applications in petrochemical industries [1], atmospheric science [2,3], clinical diagnosis and monitoring [4], etc. Gas detection can be implemented by many different approaches such as chromatographs [4], catalyst-based devices [5], semiconductors [6], and electrochemistry [7]. However, the performance may be limited by the cross responses of different gases or the noises in the amplifier circuit [5,6,7]. Currently, wavelength modulation spectroscopy (WMS) [8,9] and active intra-cavity methods [10,11] have been utilized to increase the detection sensitivity. However, the applications based on these methods are still hindered by their large system volumes, time-consuming operation, strict working environments, and complicated detection principles [12,13].

In contrast, optical fiber sensors possess advantageous features, such as inherent immunity to electromagnetic interference and to ionizing radiation, large bandwidth, compact structure, and light weight [14,15]. Therefore, they are widely utilized in industrial applications such as the distributed measurement [16,17] and the remote and harsh environment sensing [18,19,20]. The study of low-cost interrogation systems has also accelerated their practical applications [21,22,23,24].

Fiber-based gas sensing are typically realized with the combination of traditional spectroscopy techniques and optical fiber sensing systems. The properties of light, such as intensity, polarization, phase, and nonlinearity may change after the light–gas interaction around or inside the fiber, so gas species and concentrations can be obtained by detecting the variations of these parameters in fiber-based gas sensors. Nowadays, except for the widely used fiber grating gas sensors, such as fiber Bragg grating [25,26,27,28] and long period grating gas sensors [29,30,31,32,33], other fiber-based gas sensors rely on the direct absorption of molecules [34,35], Raman scattering [36,37,38], photothermal effect [17,39], photoacoustic effect [40,41,42], fluorescence [43], and surface plasmon resonance [44] have also been proposed and developed.

With the development of specialty fibers, different fibers have been applied for better sensing performance. Microfiber/nanofibers (MNFs) [45], D-shaped fibers [46,47], and photonic crystal fibers (PCFs) [16,19,48,49,50,51,52,53,54,55,56,57,58,59,60,61,62,63,64,65,66,67] have been utilized in gas sensing. MNFs and D-shaped fibers can provide light–gas interactions through their evanescent fields along the long fiber lengths, and thus they have been widely used in fiber sensing. However, the reliability of MNFs is limited by the moisture and dust in the air [68,69]. Index-guiding photonic crystal fibers (IG-PCFs) enable the confinement of light in the solid core by the modified total internal reflection, and the interaction of evanescent field and gases in the surrounding air columns. Although several sensing attempts have been made by the flexible structural designs of IG-PCFs, such as the suspended core photonic crystal fibers (SC-PCFs) [49,54,58], the evanescent fields still occupy a low power fraction of the total transmission power. The limited light–gas interaction volume in the IG-PCF cannot meet the demand for high-sensitivity gas detection. Another type of the PCF is the hollow core photonic crystal fiber (HC-PCF), which can guide light inside the hollow core by the photonic bandgap or the inhibited-coupling mechanisms. The HC-PCF can provide a large light–gas interaction overlap inside the hollow core [70,71], wide transmission bands [72,73,74], and flexible structural designs [70,72,73,74,75,76,77,78,79,80], exhibiting the potential in improving gas detection performance.

In terms of the practical application of fiber-based gas sensing, various approaches for sensing element microfabrication have been proposed for fast response of the sensor system. Various approaches of gas infiltration, such as open end-faces [19,61,63,81,82,83,84,85,86] and exposed side microchannels [16,87,88,89,90], combined with assisted driven pressure [20,36,61] can effectively shorten the diffusion time of gas and the response time of gas detection. Remote gas detection [19,20,61] and distributed gas sensing systems [16,17,39,89,91,92,93] are also realized with the well-developed microfabrication approaches.

One of the most promising fiber-based gas sensors is based on the mid-infrared (MIR) absorption spectroscopy of hollow core antiresonant fibers (HC-ARFs), which combines the advantages of strong absorption line strengths of molecules in the MIR spectral region [18], the unique fiber properties [67,78,94,95] of large hollow cores, low transmission losses in the MIR region, and inhibited mode coupling, enabling fast and sensitive gas measurements.

In this paper, we firstly introduce different principles of optical fiber gas sensing, including absorption spectroscopy, Raman scattering spectroscopy, photoacoustic spectroscopy, and photothermal spectroscopy. Secondly, the development of optical fiber gas sensors is reviewed and the advantages of HC-PCFs in gas detection are introduced. Thirdly, the light-guiding mechanisms and properties of HC-PCFs are summarized. Fourthly, we review the methods of gas sensing configuration, including the exposed end-face and the open-side microchannel in the PCF sensing element, the remote and multiplexed multi-point gas detection, and the distributed gas sensing system. Finally, we review recent advances in gas sensing with HC-ARFs in the MIR spectral region. The review on the principles, methods, configurations, and recent research advances of HC-PCF gas sensing will promote a deep understanding of this area and accelerate the development of fiber gas sensors.

## 2. Basic Principles of Fiber Gas Sensing

### 2.1. Absorption Spectroscopy

The earliest optical fiber gas sensor was based on the absorption spectroscopy, which was proposed by Inaba et al. from Tohoku university in 1979 [35]. Relying on the ‘finger-print’ absorption lines of molecules located in the ultraviolet (UV)/visible, near-infrared (NIR), or MIR regions, different gas samples can be identified and detected [96,97].

**Figure 1:**
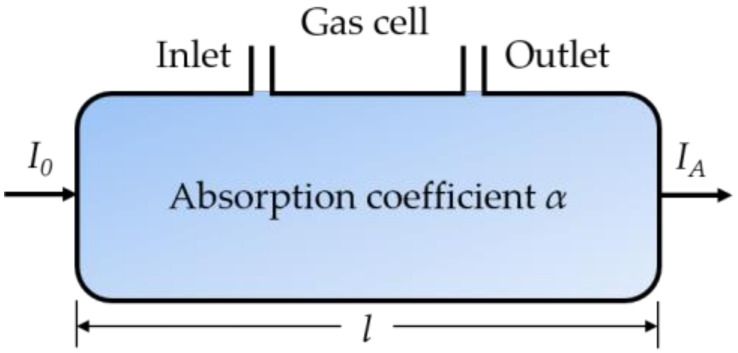
Principle of absorption spectroscopy [42].

The gas detection principle of the absorption spectroscopy is based on the Beer–Lambert law [42,50] as follows:

(1) $$I_{A}=I_{0}\exp\left(-\alpha l\right)=I_{0}\exp\left(-C\sigma l\right)=I_{0}\exp\left(-CS\varphi l\right),$$

where *I*_0_ is the incident intensity, *I_A_* represents the output intensity of light after the interaction with gas sample, *α* is the absorption coefficient, and *l* is the total path length of the absorption [34] (Figure 1). The absorption coefficient *α* represents the ability of light absorption for gas species, which is derived from the gas concentration *C* multiplied by the absorption cross-section *σ*, where *σ* is the product of the absorption line strength *S* and the lineshape function *φ* (both *σ* and *S* for a specific gas can be obtained from the spectroscopic database, such as HITRAN [98]). When an optical fiber is placed in a gas cell, the gas concentration can be obtained according to the reduced intensity at the identified wavelength of the output light.

Tunable diode laser absorption spectroscopy (TDLAS) based on the molecular absorption is widely used for trace gas sensing with the development of the diode laser [99]. The narrow linewidth and the controllable current-dependent wavelength of the laser can ensure the accurate and convenient measurement at specific wavelengths by adjusting the injected current or working temperature of the diode laser [100]. Once the laser wavelength is modulated to cover the absorption line of the target gas, the power attenuation will occur, and the concentration of gas can be subsequently retrieved by comparing the measured spectrum with the reference spectrum calculated from the spectroscopic database [67].

In a practical setup, when implementing the gas detection based on the gas absorption, an additional sinusoidal signal at a frequency *f_m_* is often used, and the trace gas concentration can be retrieved from the second harmonic component (at 2 × *f_m_*) of the photodetector signal, which is known as the WMS technique [67]. It can significantly enhance the signal-to-noise ratio, since the noise at other frequencies is not analyzed [42]. Besides, since the 2 × *f_m_* signal at the absorption center is proportional to the gas concentration, WMS is a convenient approach for the continuous monitoring of gas [67].

**Figure 2:**
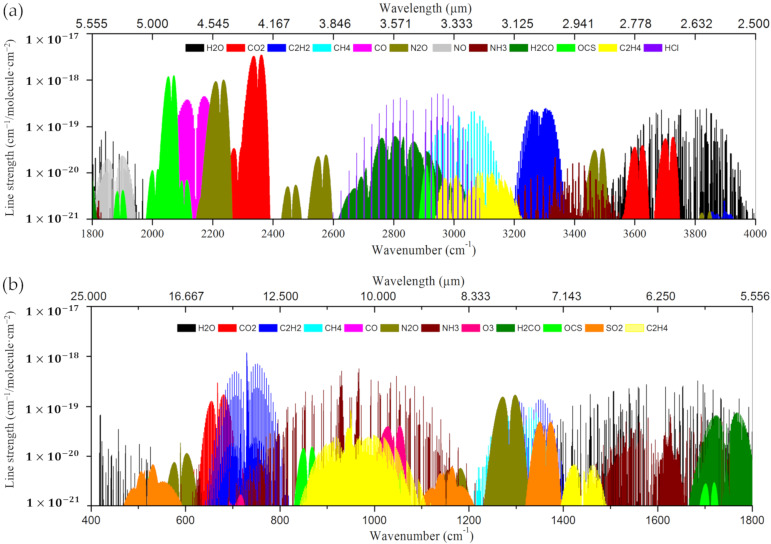
Mid-infrared (MIR) absorption spectroscopy for major atmospheric species in the wavelength range of (**a**) 2.5–5.555 μm and (**b**) 5.556–25 μm [102,103].

Due to the well-developed telecom fibers and related devices, benefited from the popularity of optical communication, gas detection in the NIR region has been extensively studied. Table 1 shows the specific wavelengths and the strengths of absorption lines in the NIR region of various gas molecules [18,34,101], and Figure 2 is the absorption spectra of major atmospheric gases in the MIR region. Actually, the line strengths in the NIR region are typically 2–3 orders of magnitude weaker than those in the MIR region [18,102,103]. For example, the line strength of methane at 1330 nm is 2 orders of magnitude weaker than that at 3310 nm. However, attention has been focused on the weaker overtone bands in the NIR region, since the lasers and detectors in this region are lower cost and more efficient. Despite this compensation, the intrinsic low absorption strength of the gases in the NIR region may still hinder the ultra-high sensitivity gas detection. More recently, with the development of MIR optics, MIR fiber gas sensing has attracted much attention and has shown great potential for high-sensitivity measurement [64,65,66,67,80,94,100,104,105,106,107,108].

### 2.2. Raman Spectroscopy

Raman spectroscopy, a technique can provide ‘fingerprint’ information about specific molecules based on their own vibrations [109], is a suitable candidate for selective multi-gas detection and has been considered as an important technique for gas sensing [37].

When the laser propagates through a gas cell, it interacts with gas, and the scattering light can be generated. The Raman scattering intensity can be expressed by a simplified equation [37]:

(2) $$I_{R}\left(\tilde{\nu_{R}},\tilde{\nu_{0}}\right)=\eta I_{0}n\left(\frac{d\sigma \left(\tilde{\nu_{R}},\tilde{\nu_{0}}\right)}{d\Omega }\right)\Omega L_{e},$$

where *I*_0_ represents the intensity of the incident exciting laser, *I_R_* is the intensity of the Raman scattering light, *n* is the molecule density of the detected gas (and it is proportional to pressure and temperature according to the ideal gas law), (*dσ*)/(*d*Ω) is the absolute differential Raman scattering cross section, Ω is the solid angle of the collected signal, and *L_e_* is the effective interaction length in fiber sensing [37]. Besides, *η* is a factor that represents the detection efficiency of the experimental setup, which depends on the optical coupling efficiency, the quality of optical components, and the quantum efficiency of the detector [37].

In the traditional free-space experimental setup, the Raman scattering efficiency is low due to small Raman scattering cross-section and low signal intensity [109,110]. To increase the Raman scattering intensity, the solid angle of the output scattering light and the Raman interaction length are two significant factors [36]. HC-PCFs can provide long fiber lengths and improved light intensity, which can bring an efficient light–gas interaction and enhance the collected scattering light intensity and the detection sensitivity while minimizing the fused silica Raman background [109].

### 2.3. Photothermal Spectroscopy

**Figure 3:**
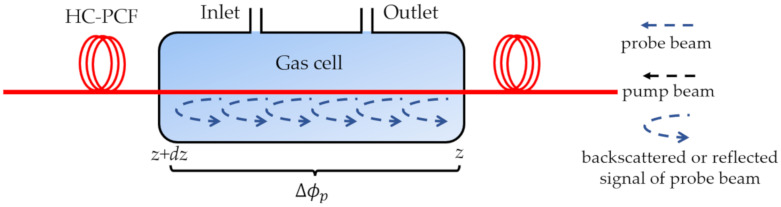
Phase modulation principle based on the photothermal effect [17].

Photothermal spectroscopy in optical fibers has been demonstrated by probing the phase change induced by the gas absorption via fiber-optic interferometry [39,89]. In a typically used pump-probe configuration, the intensity-modulated pump beam and the probe beam propagate in a gas-filled HC-PCF, as shown in Figure 3. When the standard wavelength of the pump beam is tuned to the gas-absorbed wavelength, the pump beam will be absorbed and the gas will be heated, which will modulate the local temperature, density, and pressure in the fiber. Thus, the effective refractive index, and transverse and longitudinal dimensions will change. Then, the accumulated phase of the fundamental mode of the probe beam will be modulated [89].

If the absorption is weak, the amplitude of the photothermal phase modulation over the longitudinal region [z, z+dz] can be expressed as [17]:

(3) $$\Delta \phi_{p}=P_{0}\cdot e^{-\alpha_{c}z}\cdot e^{-\alpha_{0}zC}\cdot \Delta \bar{\phi_{0}}\cdot \alpha_{0}C\cdot dz,$$

where *P*_0_ is the input peak pump power, *α_c_* is the fiber attenuation coefficient, *α*_0_ is the absorption coefficient of pure gas, and *C* is the relative gas concentration. $\Delta \bar{\phi_{0}}$ is the normalized phase modulation coefficient, representing the phase modulation magnitude of a 1-mW peak pump power in a 1-m-long fiber with 1 cm^−1^ gas absorption [17]. The coefficient is independent from the absorption intensity, and it is remarkably different for different gases and different absorption wavelengths [17]. HC-PCFs can provide a larger light–gas overlap over a long-length accumulation of phase modulation, enabling an ultra-sensitive gas sensing with a large dynamic range. It is worth noting that detection resolution can be improved by reducing the noise level when the signal is filtered with the sacrifice of the detection bandwidth. Thus, the detection limit normalized on the detection bandwidth expressed in terms of units/Hz^1/2^ is more accurate on the application circumstance of varying signals to be detected.

### 2.4. Photoacoustic Spectroscopy

**Figure 4:**
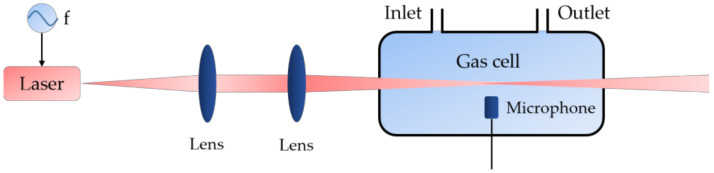
Principle of gas detection based on the photoacoustic spectroscopy. f: periodic modulation with a frequency f [42].

Photoacoustic spectroscopy is an absorption-based technique with its relative simplicity, ruggedness, and overall sensitivity, which plays a key role in trace gas detection [40,41,42]. Figure 4 illustrates the basic sensing principle of the photoacoustic spectroscopy. The laser with periodically modulated intensity or wavelength propagates to the gas cell, where the laser is absorbed by gas molecules and converted to local heating via collisions and de-excitation. Then, the acoustic waves will generate and can be detected by a microphone, and the gas concentrations can be restored by data processing [28].

## 3. SC-PCFs and HC-PCFs

In order to fully realize the potential of optical fibers in gas sensing, an optimal alternative is to enhance the interaction between the light transmitted in the fiber and the detected gases. One achievable method is to utilize the evanescent light field of MNFs [45] and D-shaped fibers [46,47] to interact with surrounding gases. For MNFs, a large fraction of the evanescent field power is in the air [45], thus allowing the light–gas interaction more efficiently over a length of a few centimeters. The D-shaped fiber can also provide an evanescent field over a much longer length [47]; however, the evanescent field in the air is relatively weak, and the light–gas interaction is not efficient. For the above two types of fibers, the presence of moisture and dust can cause severe degradation when they are exposed in the air for a long time [68,69], such as the decrease of the transmission efficiency and the degradation of the mechanical strength in practical applications.

To meet these challenges, the attention has been transferred to the PCFs with air hole structures. The IG-PCF, with a cladding consisting of air holes and a solid core that runs along the entire fiber length, is capable of confining light in the core by modified total internal reflection and has created new possibilities for the interactions of optical field and gases. In 1999, Monro et al. theoretically proposed a holey fiber gas sensor based on the direct absorption spectroscopy (DAS) [48], indicating the superiority of such fibers in gas sensing. In 2001, Hoo et al. used a 75-cm-long IG-PCF to detect acetylene in experiments [49], achieving a detection limit of about 5.5% compared with the direct absorption cell for the same interaction length, and indicating the evanescent field power located in the air holes for this fiber was about 5.5% of the total power carried by it. Furthermore, to characterize the response time and the relative sensitivity of the gas sensing model, the gas diffusion dynamics was presented in 2003 [50], and a PCF-based acetylene sensor with periodic windows was proposed [50]. The preliminary simulation and experiment results showed that the acetylene sensors can achieve a response time of about 1 min and a detection limit of lower than 6 parts per million (ppm).

**Figure 5:**
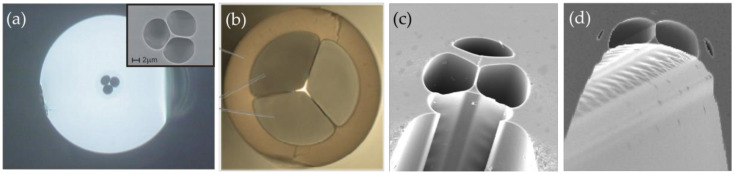
(**a**,**b**) Cross-section images of suspended core photonic crystal fibers (SC-PCFs) with triangular-like microcores [53,58]. (**c**,**d**) Cross-section images of exposed-core SC-PCFs fabricated by etching the drawn fiber and drawing from the drawing tower [112]; the image sizes are 40 µm.

Subsequently, the SC-PCF gas sensor has been developed for the simpler structure. As shown in Figure 5a, this fiber has a small core in the wavelength or sub-wavelength scale and a holey cladding with a large air filling fraction [49,54]. To improve the sensing sensitivity, the power fraction of the evanescent field in the air holes could be increased to more than 20% of the total power by adjusting the structural parameters, such as the number and the size of air holes [49]. However, when the SC-PCF is connected to a conventional single mode fiber (SMF), the fusion splicing loss is large due to the mode field mismatch and the air hole collapse in the PCF. To overcome this problem, a compact and contamination-free SC-PCF gas microcell was fabricated by post-processing an endless single-mode PCF with the mode field diameter similar to a conventional SMF [58]. The PCF was tapered at a chosen location with high pressure in the selective holes, the selected air-columns were expanded, and a SC-PCF microcell was fabricated, as shown in Figure 5b. It is worth noting that the holes at the fiber ends were used for the infiltration of the ambient gas in the sensing process.

In order to reduce the response time, the gas to be detected can diffuse into the evanescent field area by drilling holes on the outer cladding [16,52,57,58] or by opening slots on the side [51,55,111,112]. Smith et al. reported two new methods for fabricating exposed core fibers in Ref. [112]. The first method was to etch the outer cladding of fiber (Figure 5c), and the other method was to draw the fiber from the structured preform (Figure 5d).

In addition, other PCF gas sensors based on the evanescent field have been proposed in recent years. For example, a side-channel PCF was proposed as a high-sensitivity surface-enhanced Raman scattering sensing platform in Ref. [113], a depressed-index core PCF was designed for gas sensing applications in Ref. [114], a microstructured core PCF was fabricated with polymer materials to improve the detection sensitivity in Ref. [115], and a C-type fiber was used to provide gas inlet and outlet channels to realize the all-fiber gas sensing in Ref. [116]. Optical fiber gas sensors based on the evanescent field can provide an extremely long length for the light–gas interaction and for the distributed fiber sensing. However, the evanescent field occupies a low power fraction of the total transmission power. HC-PCFs, with the capability of guiding light in the central hollow core [71,117] by designing the microstructure claddings, provide a better platform for the light–gas interaction than index guiding fibers based on the evanescent field [81,118]. Specifically, the key advantages of HC-PCFs in gas sensing are listed as follows [56,61,119,120]:

- Up to 99% of the total transmission power can be confined in the hollow core [70], improving the sensing sensitivity.
- The light–gas overlap in the fiber is significantly increased, and thereby the fiber length can be greatly reduced, improving the response speed.
- A wide transmission wavelength band can be chosen from the deep ultraviolet (UV) to the MIR region [72,73,74] through the flexible structure design of HC-PCFs. Especially, the MIR region is very suitable for gas sensing based on the gas absorption that was mentioned in Section 2.
- The light confined in the core is less affected by the fiber material in the cladding. Furthermore, HC-PCFs possess advanced features of low nonlinearity, short delay, high damage threshold, and good thermal stability.

## 4. Structures and Properties of HC-PCFs

HC-PCFs have many useful advantages in gas sensing. In order to make full use of these fibers, it is necessary to have a deep understanding of their light-guiding mechanisms, e.g., photonic bandgap guiding [56,61,62,70,76,81,118,119,120,121,122,123], anti-resonant guiding, or inhibited-coupling (IC) guiding [75,77,78,79,124,125,126,127,128,129,130,131,132]. In terms of fiber materials, the most popular material is fused silica [72,73,132,133]. Polymer [127,134] and soft glass, such as chalcogenide glass [135] and fluoride glass [136] are also used.

**Figure 6:**
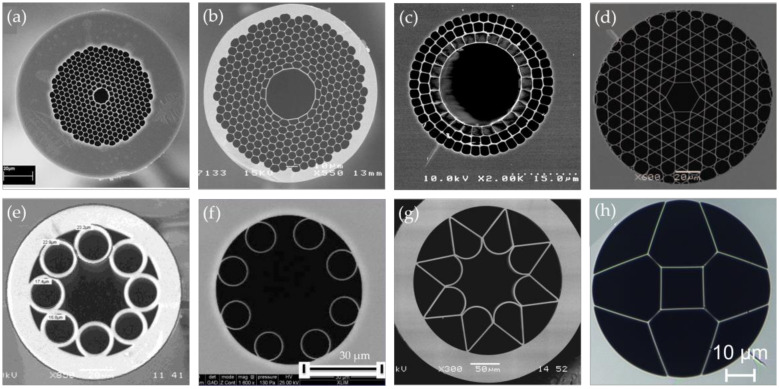
Scanning electronic microscopy (SEM) images of some representative hollow-core photonic crystal fibers (HC-PCFs): (**a**) Commercial available hollow core photonic bandgap fiber (HC-PBGF): HC-1550-02 from NKT Photonics [138]; (**b**) HC-PCF designed for the guiding in the MIR region [137]; (**c**) Hollow core Bragg fiber [74]; (**d**) Kagome lattice HC-PCF [124]; (**e**) hollow core antiresonant fibers (HC-ARF) [59]; (**f**) nodeless HC-ARF [78]; (**g**) hollow fiber with negative curvature of the core wall [73]; (**h**) double anti-resonant hollow square core fiber [72].

Figure 6a is a hollow core photonic bandgap fiber (HC-PBGF) composing of honeycomb air holes lattice in the background of silica, and the periodic lattice structure forms photonic band gaps. Thus, when the light frequency is within the photonic bandgap, the light is not allowed to propagate in the cladding, and it is confined to the defect region of the low refractive index, the central air core [71,118]. Due to the low transmission loss and the rich commercial products [81,123], HC-PBGFs have been widely investigated since their first successful fabrication in 1999 [121]. The transmission wavelength bands for the most widely used commercial HC-PBGFs are in the visible and the NIR regions [70,71,117]. Studies have shown that the drawing of HC-PBGF with a small air hole pitch can effectively cause a blue shift for the transmission window. However, the fabrication of PCFs with a small air hole pitch is challenging [78]. In addition, the inherent transmission bandwidths of the fibers are narrow, which hinders their applications in guiding broadband light [124]. The fundamental mode and the surface mode are easy to couple, and a small part of light is still in the silica, which limits their applications in guiding the UV and the MIR light [124]. In order to improve the performance of HC-PBGFs in the MIR region, a silica-based HC-PBGF (Figure 6b) with a transmission bandgap above 3 μm was reported in Ref. [137]. The peak of the bandgap was at 3.14 μm with a typical attenuation of 2.6 dB/m. In addition, more novel HC-PBGFs guiding in the MIR band can be fabricated [135,136] by utilizing the characteristics of the chalcogenide glass and the fluoride glass.

The other type of HC-PBGF is hollow core Bragg fibers, as shown in Figure 6c, which consists of concentric cylindrical silica rings separated by nanoscale support bridges [74]. The ring structure in the cladding is similar to a multilayer Bragg reflector, where the hole rings are averaged in the azimuthal direction to form a low refractive index layer, and the solid regions between the aperture rings form high refractive index layers [76,134]. Therefore, this structure supports the photonic band gap in the radial direction and can confine light in the hollow core [139]. Its transmission window is located in the NIR range, and the transmission window of the Bragg fiber can be shifted to the MIR or even far-infrared range [74] by increasing the overall size of the fiber microstructure. Besides, considering the attenuation loss of silica glass, researchers have proposed the utilization of a low-loss polymer material instead of silica glass to achieve a more efficient hollow core light guiding at longer wavelengths [74], and hollow core Bragg fibers based on semiconductor glass and polymer [122] have shown great potential for MIR transmission.

The mechanism of the IC guiding HC-PCFs is inhibited coupling between different modes. When the coupling between the core mode and the cladding mode is inhibited, the light that is guided in the core can propagate along the hollow core with a low transmission loss [126,127]. The IC guiding HC-PCFs exhibit better performance than HC-PBGFs in terms of the transmission bandwidth, but they tend to suffer higher losses [125]. A Kagome lattice IC guiding HC-PCF is shown in Figure 6d, with a cladding that consists of fine silica meshes arranged in a Kagome lattice [75]. This Kagome lattice HC-PCF structure can support a wider transmission band by the structure design, even including the band in UV, visible, and infrared ranges [75,124,130].

Another type of the IC guiding HC-PCF, also known as a HC-ARF, is shown in Figure 6e, with a ring of periodically-arranged silica capillaries located in the cladding [77,79,132]. Light can be confined within the hollow core by the anti-resonant effect [131], and it can be enhanced by the inhibited coupling between the core mode and the cladding mode [78]. The transmission loss of 11 dB/m for a 10.6 μm CO_2_ laser in a chalcogenide glass fiber with a negative curvature was demonstrated by optimizing the cross-section structure [135]. In particular, as shown in Figure 6f, a nodeless HC-ARF shows a lower confinement loss when the cladding capillaries are not in contact [78]. By optimizing the design of the tubular cladding and using thinner glass tubes, Debord et al. [78] reported several fabricated low-loss single-ring tubular HC-ARFs that guiding light in the UV and the NIR ranges, including one sample with a record transmission loss of 7.7 dB/km at approximately 750 nm and another sample with an ultrabroad fundamental band with loss in the range of 10–20 dB/km, spanning from 600 to 1200 nm.

In addition, novel HC-ARFs were designed and fabricated with UV or MIR transmission bands. For example, a negative-curvature hollow-core silica fiber (Figure 6g) for MIR transmission had a minimum attenuation of 34 dB/km at the wavelength of 3050 nm [73]. This negative curvature core could even achieve a transmission band of more than 4 μm. A double anti-resonant square-core silica fiber (Figure 6h) was designed with a square core formed by very thin silica strands and four comparably large air holes around it [72], showing different transmission windows from the deep UV region to the NIR region. The anti-resonance between its core mode and adjacent mode forms a new modified tunneling leaky mode for low loss transmission.

The hollow core of HC-PCF provides a perfect platform for light–gas interaction. Compared with HC-PBGFs, IC guiding HC-PCFs have the excellent ability of adjusting the desired transmission band from the UV range to the MIR range by a flexible and simple structure design according to the different response wavelengths of detected gases.

In order to explore the potential of HC-PCFs in gas sensing and improve sensing performance, on the one hand, researchers have studied the different structural and material designs of HC-PCFs. In addition to the above-mentioned HC- PCFs, other HC-PCFs have been designed for the target needs [63]. On the other hand, various HC-PCF gas sensing configurations, such as sensing elements [36,38,52,60,61,62,63,81,82,83,84,85,87,88,105,120,140,141,142,143,144,145,146,147], remote and multiplexed multi-point detections [19,20,86], and distributed gas sensing systems [16,17,39,89,91,92,93] have been proposed. With the development of gas infiltration approaches, the response time of the gas sensor is reduced, and the sensing performance is further improved. The detections of common harmful gases, such as methane [36,62,81,82,83,105,146], acetylene [20,61,85,120,145,147], ammonia [60,82], carbon dioxide [61], hydrogen cyanide [82], and hydrogen [38] have been demonstrated.

## 5. Configurations of Gas Sensing in HC-PCFs

### 5.1. HC-PCFs with Gas Infiltrated from Fiber Ends

A simple way to introduce detected gases into the HC-PCF is filling the gases through the fiber end [81,82,83,84]; for example, one end of the HC-PCF is exposed in a gas chamber or in an ambient environment. Generally, the exposed end of the HC-PCF is butt-coupled to the conventional fiber with a suitable gap for gas infiltration, while the other end of the HC-PCF is spliced with the conventional fiber for light launching. The all-fiber configuration can improve the efficiency and compactness of measurement system. For instance, various gas detections, such as acetylene, hydrogen cyanide, methane, and ammonia were implemented in a HC-PBGF based on the DAS by Hansen et al. in 2004 [82]. One end of the HC-PCF was connected to the laser source via the conventional fiber, while the other end of the PCF was placed in a gas chamber, coupled to the multimode fiber (MMF) in a V-shaped groove, and the MMF output was connected to the detector to obtain light intensity at different wavelengths. The gas chamber can provide the gas to be detected, and the coupling gap of about 50 μm in the V-groove can achieve a reasonably high coupling efficiency and an efficient infiltration.

**Figure 7:**
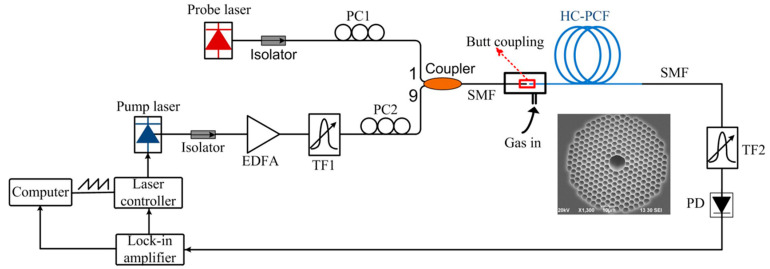
Experimental setup for the gas detection with a 15-m-long HC-PCF. The inset shows the SEM image of the HC-PCF [38].

In 2017, Yang et al. reported an all-fiber hydrogen sensor based on the continuous wave stimulated Raman gain spectroscopy using a similar gas-filling configuration (Figure 7) [38]. The experiment showed that the detection limit could be reduced to 17 ppm within an average response time of 250 s. This all-fiber system operating in the communication wavelength band provided effective and compact trace gas detection with high sensitivity and high resolution.

Buric et al. have implemented the spontaneous gas phase Raman scattering measurement using an HC-PBGF in 2008 [36]. The light-input end of PCF was exposed in the free space, while the other end of the PCF was coupled to an MMF in a V-groove placed in a high-pressure chamber as the gas inlet position. Significantly, for the Raman scattering in the HC-PCF, the light–gas interaction is enhanced, since the light is mainly located in the core [37], and the Stokes wave was efficiently generated and transmitted along the hollow core. Due to the long interaction length and large mode-field overlap in gas samples, the HC-PCF structure can provide two orders of magnitude enhancement of the Stokes power compared with that in the free-space configuration. The excellent performance was verified by the enhanced Stokes signal of nitrogen in Ref. [36].

Different gas species, such as nitrogen, oxygen, carbon dioxide, toluene, acetone, and 1,1,1-trichloroethane were subjected to the gas Raman detection with a 30-cm-long HC-PBGF, referring to the ambient nitrogen Raman signal in 2012 [86]. One end of the PCF was placed under the Renishaw Invia Raman system, and the distant end of the PCF was placed in the surrounding environment or in a sealed container for remote sensing. In the sealed container, a tiny droplet was added to get the corresponding vapor at a certain concentration. The achievable sensitivity was increased by about 700 times compared with the detection using the bulk Raman detection.

**Figure 8:**
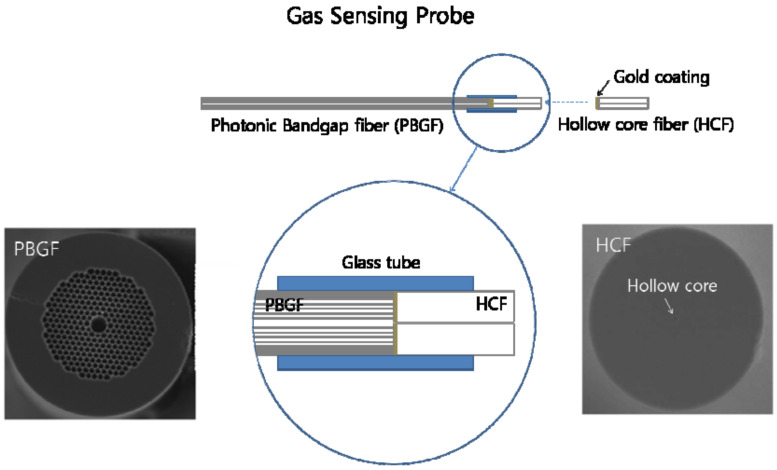
Schematic structure of the gas-sensing probe with an replaceable insert [20].

In 2012, Lim et al. fabricated a remote gas sensor with a replaceable fiber probe based on the gas absorption spectroscopy [20]. As shown in Figure 8, the probe consisted of a glass tube inserted by an HC-PBGF with a core diameter of 10.9 μm and an end-coated hollow core fiber (HCF) with a core diameter of 2 μm. The HCF was designed to act as both a gas gate to the PCF and a mirror to reflect the guided light back to the PCF. At the other end of the probe, the PCF was coupled to the SMF in a side-drilled fiber connector to control the gas pressure in the PCF. Besides, in order to reduce the Fresnel back reflection from the end face of the SMF, the coupling end of the SMF was prepared with an 8° angle. Utilizing this gas sensor, the detection of acetylene with 10% concentration presented strong absorption at the wavelength of 1510–1540 nm under different driven pressures. This compact fiber sensing probe exhibited a potential in remote and harsh environment.

In 2009, Carvalho et al. proved that the gas diffusion rate in the PCF with two open ends exposed in the gas cell was much faster than that with one open end [19]. Subsequently, a variety of double-end fed HC-PCF sensing units and remote gas sensing systems have been implemented. Wynne et al. [61] proposed a HC-PBGF sensor for the detection of multicomponent gas samples based on the absorption spectroscopy, showing a short response time. For example, with a driven pressure of less than 15 psi in the 2-m-long commercially available PCF, the detection of acetylene with a concentration of less than 100 ppm was achieved in a sub-minute time scale [61].

Kassani et al. designed and fabricated an evanescent field gas sensor based on a novel suspended ring core PCF in 2015 [63]. The proposed gas sensor unit consisted of MMFs connected to the light source and the detector, suspended ring core photonic crystal fiber as a sensing medium, and C-type fibers as fiberized inlet/outlet components. A clear weak absorption line was observed with the 25-cm-long PCF filled with acetylene gas, demonstrating improvement in both sensitivity and response time.

In 2017, Tan et al. proposed a high-finesse HC-PBGF resonating Fabry–Perot gas cells for gas detection [84]. In the configuration, a section of PCF was mechanically coupled to two sections of SMFs with mirrored end faces through slight gaps to form a Fabry–Perot absorption cell. The slips in the mechanical connectors and the slight gaps between the fibers allowed the gas to flow. Since light would pass through the gas many times as it traveled back and forth within the cavity, the effective absorption path length and the cavity fineness were both increased. Compared with the single-pass and non-resonant PCF cell, this resonant PCF cavity can significantly reduce the influence of modal interference on gas detection and improve the detection sensitivity [84]. Therefore, a detection limit of 7 ppm for acetylene was achieved using a 9.4-cm-long Fabry–Perot fiber gas cell.

With two ends open, multi-segments of HC-PCFs can be connected mechanically to improve the sensitivity. Gayraud et al. carried on the methane detection based on the Fourier transform infrared spectroscopy and achieved the detection limit down to 50 ppm, utilizing an HC-PBGF whose transmission bandgap covered the fundamental absorption lines of methane in the wavelength range of 3.31–3.32 μm [83]. They used fragmented coupled PCFs to shorten the gas diffusion time from several minutes or hours to only a few seconds. Besides, the optimal coupling gap between the fibers was adopted to minimize the coupling loss of less than 0.1 dB per gap.

**Figure 9:**
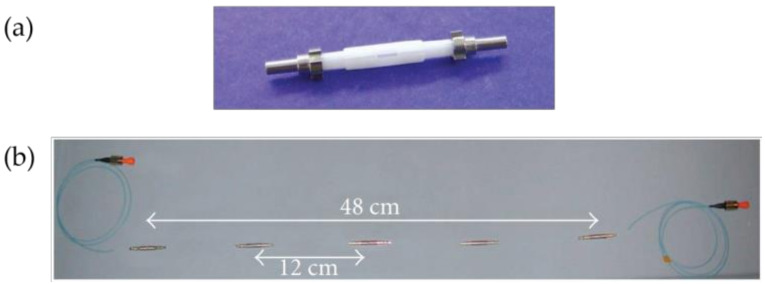
(**a**) Fiber section connector. (**b**) Multi-section sensing head containing 4 sections of photonic crystal fibers (PCF) [19].

Carvalho et al. developed a portable gas sensing system for the remote trace methane sensing with the HC-PBGF [19], which was able to interrogate four remote sensing units simultaneously based on the WMS technique. The sensing unit has two open coupling ends exposed in the gas cell, leading to a response time of approximately 248 s to achieve 95% of the steady state, and permitting a methane detection limit of about 158 ppm. In order to optimize the sensitivity of the sensor without compromising the response time, a structure was designed with periodic openings along the fiber axis. As shown in Figure 9, several sections of PCF were aligned by the connectors. However, the superficial modes at the boundary of the fiber hollow core would induce interference effects, reducing the signal-to-noise ratio [19].

**Figure 10:**
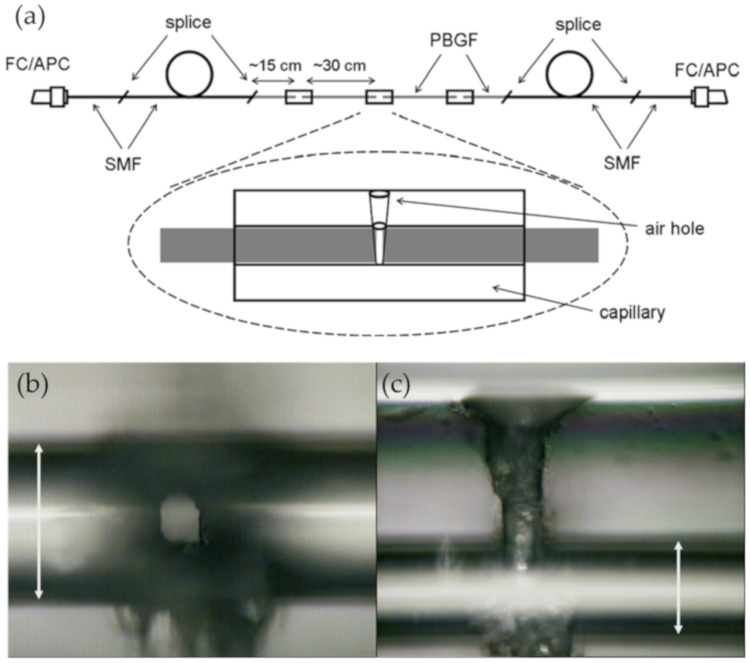
(**a**) Fiber assembly for the gas sensor. FC/APC: ferrule connector/asperity polishing connector. (**b**,**c**) Images of the laser drilled hole in a glass capillary tube side, from a vertical and a front view respectively [85].

Parry et al. [85] designed a gas sensor with four separate HC-PBGF sections. As shown in Figure 10, the PCF sections were butt-coupled with gaps inside glass capillaries, and the glass capillaries were drilled with holes (Figure 10b,c) on the side by a pulse laser. The ends of PCF were located under the holes and separated by a small gap with the order of 10 μm, which opened the hollow core of the PCF to the surrounding and allowed the gas to flow. This sensor has been used for the detection of methane and acetylene, and the lowest detectable acetylene concentration was 0.05%.

The above methods to fill HC-PCFs from end faces are convenient to implement. Especially, two or more open ends with a driving pressure can effectively improve the gas diffusion efficiency and reduce the response time for gas sensing. However, the response time in a long-length HC-PCF may not be very short, since the filling time is proportional to the square of the ratio of the fiber length to the core diameter [141,148]. The two-fiber connection with a gap will inevitably introduce an extra loss. In addition, the mechanical connection may be sensitive to the external vibrations and ambient temperature in practical applications.

### 5.2. HC-PCFs with Gas Access from Fiber Sides

When implementing the distributed gas sensing based on HC-PCFs, it is necessary to interrogate the gas sample over the entire fiber length, while the exposed end faces can only collect gas from one point or few points [16]. Besides, the response time of the device is significantly important for its practical application. As a result, scholars have devoted their energy to study new methods to fill gas into the hollow core via drilling holes through the fiber side. The side microchannels are often introduced by the focused ion beam (FIB) [88,143,146] or the pulsed laser [16,17,39,52,85,89,91,92,93,120,144,145,147]. In addition, the microchannels on the fiber side can be fabricated by the arc discharge under pressure [87].

**Figure 11:**
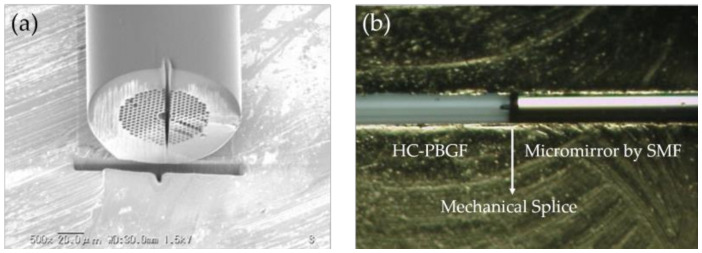
(**a**) SEM image of a penetrated hole of 5 × 5 μm^2^ in an HC-PBGF. (**b**) Image of the mechanical connection between the PCF and the SMF with a micromirror [88].

In 2011, Li et al. fabricated a HC-PBGF gas cell with a double optical path length [88]. The PCF was drilled by the FIB to open a side channel (Figure 11a). The mirrored end of the SMF was connected to the PCF mechanically as shown in Figure 11b, doubling the optical transmission path without changing the gas flow. Based on the gas absorption spectroscopy, a detection limit of 20 ppm for ammonia was achieved.

Lehmann et al. reported the open side channel that penetrated the cladding structure in the HC-PBGF in 2005 [16], indicating that this structure can be used in the distributed gas sensors. Several fiber samples were drilled using an ArF-laser (193 nm) with a repetition rate of 50 Hz, and an example of the radial drilling of a polydimethoxysilane (PDMS) coated HC-PCF was presented.

**Figure 12:**
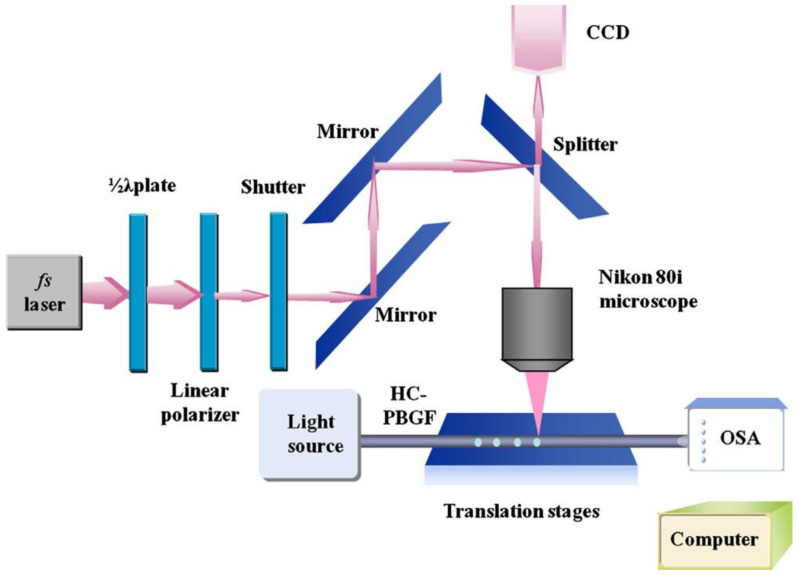
The femtosecond (fs) laser system for fabricating microchannels in an HC-PBGF [42].

Figure 12 shows a femtosecond laser setup [93] for the microchannel fabrication on the side of the HC-PBGF. The fiber was mounted on a three-axis translation stage controlled by the computer. The femtosecond laser was firstly focused and aligned to the fiber surface, and then its focus point moved toward the fiber core. This process can be repeated several times to complete multiple gas microchannels from the fiber surface to the fiber core.

The controllable micro-drilling techniques have been utilized in a number of studies on the gas sensing with side-open HC-PCFs. In 2007, Hensley et al. [144] implemented the femtosecond laser drilling technique to prepare a pressured HC-PBGF gas cell, estimated the drilling losses, and measured the acetylene spectroscopy. Six evenly spaced holes were drilled over a 2-mm-long section of a 33-cm-long PCF. The total loss due to drilling was about 2.1 dB at the wavelength of 1550 nm, which means the drilling loss was 0.35 dB for a single microchannel.

The periodic microchannels were drilled in a HC-PBGF gas cell by a femtosecond Ti:sapphire laser in 2010 [91]. Based on the DAS, a fast-response methane sensor was realized. For the sensing fiber with a whole length of 7 cm, seven side holes with the space of 1 cm along the fiber were introduced, realizing a diffusion response time of 3 s and a detection limit of 647 ppm. In addition, since the total insertion loss of the seven side holes was only 0.5 dB at the wavelength of around 1650 nm, it was advised to fabricate a long-length (up to tens of meters) fiber gas sensor with hundreds of holes without compromising the response time to achieve a high-performance distributed sensing [91]. However, the response time was related to the number of holes and the distance between holes, and the researchers indicated that a 1-m-long fiber required approximately 100 drilled channels to maintain a fast response [76].

In 2014, Yang et al. studied the effect of intermodal interference on the performance of the HC-PBGF gas sensing [120]. The input end of the 13-m-long PCF was mechanically coupled to a SMF, and the authors have demonstrated that a short gap between them could result in an interference signal in the recorded spectrum, which was believed to be the mode interference between LP_11_ and LP_21_ modes due to the lateral offset between the cores of the two fibers. They also calculated the mode launch amplitude for a lateral offset with 2 and 3 μm for different longitudinal gaps and verified this point [120]. Both experiments and calculation [120] have shown that the launch amplitudes of higher order modes LP_11_ and LP_21_ could be decreased significantly for the gap from 0 to 100 μm but very little from 100 to 150 μm. Thus, considering the coupling loss at the joint, an optimal gap of 100 μm was chosen to act as a gas-fed channel with a minimum mode interference while maintaining a reasonably low loss. The output end of the PCF was spliced with another SMF, and a microchannel was drilled near the splicing joint using an 800 nm femtosecond laser. They utilized the WMS technique with appropriate modulation parameters, achieving the detection of acetylene with a detection limit of less than 1 ppm.

Most of the above HC-PCF gas sensors are based on the laser absorption spectroscopy to identify and detect trace gases through the molecular ‘fingerprint’ absorption lines. However, the low-loss transmission window of the silica fibers is located in the wavelength range of 0.5–1.8 μm [89], where there are only the relatively weak molecular harmonic absorption lines, greatly reducing the detection sensitivity [89]. To achieve significantly high sensitivity, a long length HC-PCF is necessary, but it usually means the increase of the response time.

An all-fiber gas sensor based on the photothermal interferometry (PTI) was proposed by Jin et al. [89]. This sensor utilized a HC-PBGF guided the NIR light to detect trace gases with a large dynamic range, achieving an extremely high sensitivity. Instead of directly measuring the attenuation spectroscopy after the gas absorption, the photothermal effect in HC-PCF was used to detect the phase change caused by the gas absorption in the fiber interferometry. For the drilling experiment, 15 microholes were drilled by an 800 nm femtosecond laser, with the diameter of 5 μm and the space of 2–7 cm along the fiber. The average loss per microchannel was estimated to be 0.02 dB in the wavelength range of 1500–1600 nm, and the total insertion loss was 4 dB. Thus, the authors have demonstrated an all-fiber acetylene gas sensor with a detection limit of 2 parts per billion (ppb) level.

Drilling holes along the HC-PCF length can significantly reduce the response time; however, when more microchannels are needed to improve the response, there are several shortcomings such as a time-consuming drilling process, high insertion loss, and modal coupling fluctuations. For example, Yang et al. investigated the impact of drilling microchannels on the transmission loss and mode interference [147]. The study has shown that for a 2.3-m-long HC-PBGF sample, the average loss in the wavelength range of 1525.5 to 1535 nm for 80 microchannels was measured to be around 0.78 dB, and for the 3.2-m-long HC-PBGF sample, the total loss of the microchannels was around 3.9 dB for 144 microchannels [147]. The mode coupling was also observed when the number of drilled holes was more than 48; as a result, the transmitted light intensity fluctuated and the signal to noise ratio decreased.

**Figure 13:**
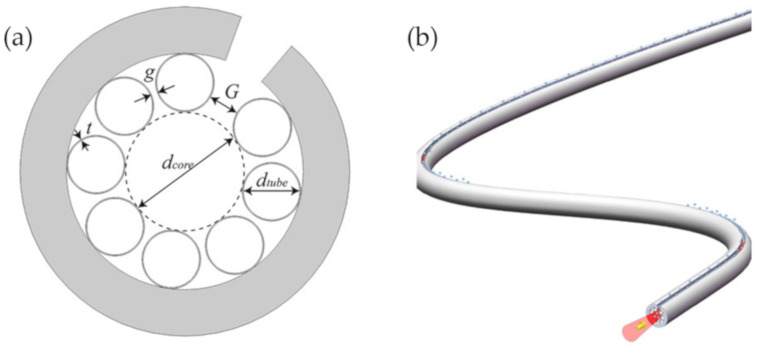
(**a**) Cross-section of the exposed-core unsymmetrical-gap nodeless HC-ARF. (**b**) Schematic three-dimensional image of the PCF with an exposed core, where the light interacts with matter [80].

Recently, Hao et al. [80] have proposed an exposed-core unsymmetrical-gap nodeless HC-ARF, providing a novel way to achieve ultrafast and sensitive gas sensors. As shown in Figure 13a, the exposed-core HC-ARF possesses a large unsymmetrical gap among the cladding tubes, and a side slot on the cladding aligning with this large gap. Figure 13b shows a three-dimensional image of this PCF with an exposed core, which brings a great convenience to shorten the response time to less than 1 ms. Micro-processing of the outer cladding, such as a lateral slit, can provide an elegant channel along the fiber, and it will not affect the inner cladding structure. Thus, the property of the confined light, and the transmission loss will not be affected. The special HC-ARF is very useful for a variety of applications that require the lateral channel.

The fabrication of this fiber can be achieved from fiber drawing process or fiber microcutting post-process without resulting in operation difficulty and extra losses. For the fiber microcutting post-process, the larger gap provides an abundant space for the operating of laser or polish to obtain a lateral microslot along the entire fiber length.

**Figure 14:**
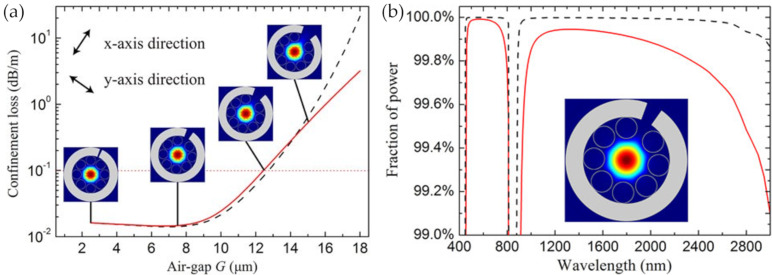
(**a**) Confinement losses in two orthogonal polarization directions (the *x*-axis is the black dashed line and the *y*-axis is the red solid line) as functions of the larger gap G for the PCF at the wavelength of 1550 nm. The inset are the fundamental mode patterns in the *y*-axis direction with G values of 2.5, 7.5, 12.5, and 15 μm at the wavelength of 1550 nm, respectively. (**b**) A fraction of power inside the air core (red solid line) and in air (black dashed line) of the fiber with a G value of 7.5 μm. The inset is the mode pattern of the fundamental mode at the wavelength of 1550 nm [80].

The structure parameters, especially for the gap parameters, have been optimized. The desired mode birefringence, mode pattern, and fraction of power in the core with low confinement loss were achieved by the analysis of the full-vector finite element method. The symmetrical tube gap in Figure 14a is 2.5 μm, and the core diameter is 41 μm. Then, two orthogonal polarization directions were defined to optimize the unsymmetrical gap, where the direction parallel to the fiber microslot was defined as the *x*-axis direction, and the vertical direction was defined as the *y*-axis direction. In general, when the larger gap size G is less than 10 μm, the confinement losses in two polarization directions are similar. With the increase of G, the confinement loss in the *x*-axis direction will increase faster than that in the *y*-axis direction, because when the large gap increases over a certain extent, the light will leak out through it. Specifically speaking, when G is less than 13 μm, the confinement loss is less than 0.1 dB/m, but the confinement loss will increase rapidly when G continues to increase. The insets in Figure 14a are the fundamental mode patterns of the designed fiber with different larger gap sizes (2.5 μm, 7.5 μm, 12.5 μm, and 15 μm in the optimization range) at the wavelength of 1550 nm, and the confined light begins to leak out from the larger gap when G is 15 μm.

Figure 14b shows the fractions of power in the fiber core and in air as functions of wavelength when G is fixed to 7.5 μm. The fraction of power over 99.85% can be confined in the core, which is even higher than that in HC-PBGFs. The ultrahigh fraction of power in air indicated the ultrahigh efficiency of the light–matter interaction when the hollow core is exposed laterally to the external environment. This exposed-core HC-ARF shows a fast response through the lateral microslot along the entire fiber length, and an ultrahigh sensitivity by the large overlap between the confined light and the infiltrated matter, predicting a high performance in fiber gas sensors.

## 6. MIR HC-ARF Gas Sensors

The study of gas sensors in the MIR region is essential for high sensitivity, since the fundamental vibration–rotation transitions of many gas molecules locate in the spectral region. Furthermore, in this region, the absorption strengths are significantly high, about 100–1000 times greater than those of the overtone lines in the NIR region [18,105], which means that the molecular absorption will be more efficient and the ultrahigh sensitivity in gas detection will be possible. In recent years, MIR laser sources, such as interband cascade lasers (ICLs) and quantum cascade lasers (QCLs) [67], which can offer high-power, tunable, and pulsed or cw radiation at wavelengths ranging from 3.5 to 19 μm [100], have greatly promoted the applications of gas sensing.

Several attempts have been made for gas detections with different fibers and principles in the MIR region [64,65,66,67,94,100,105,106,107,108]. In these studies, hollow silica waveguides (HSWs), HC-PBGFs, and HC-ARFs have been used as gas cells. HSWs consist of a silica tube with bore diameters ranging from 250 μm [106] up to 2100 μm [107], which means that it is difficult to be compatible with traditional fiber systems. Besides, they suffer from significant transmission losses and bend losses when coiled, and thus the output power is lower compared with other gas cells [100]. In terms of the application of HSWs for the MIR gas sensing, to improve the wall reflectivity in the MIR region, the inner wall needs to be deposited with silver that is then exposed to a halogen, which converts some of the silver to a silver halide [100,108]. Compared with HSWs, HC-PBGFs are generally designed to operate in the NIR region with low attenuation, and the research of HC-PBGFs in the MIR region has been rarely reported. One example is the use of a specially designed silica-based HC-PBGF to implement the methane sensing at the wavelength of about 3.2 μm based on Fourier transform infrared spectroscopy, which has achieved a detection limit of 50 ppm [105]. However, HC-PBGFs can bring strong optical fringes in the recorded spectrum from the interaction between core mode and surface modes unless complicated sensing techniques are used [149]. Besides, the hollow cores in HC-PBGFs are relatively small, resulting in a relatively long gas diffusion time (typically more than few minutes even for short pieces of fiber) [85].

HC-ARFs are suitable candidates for MIR gas detections compared with HSWs and HC-PBGFs. Since HC-ARFs feature large hollow cores [78], low losses, and broad bandwidths in the MIR region [66,67,94], and reduced couplings between the core modes and the cladding modes [149], they are expected to present better performance in MIR gas sensors.

**Figure 15:**
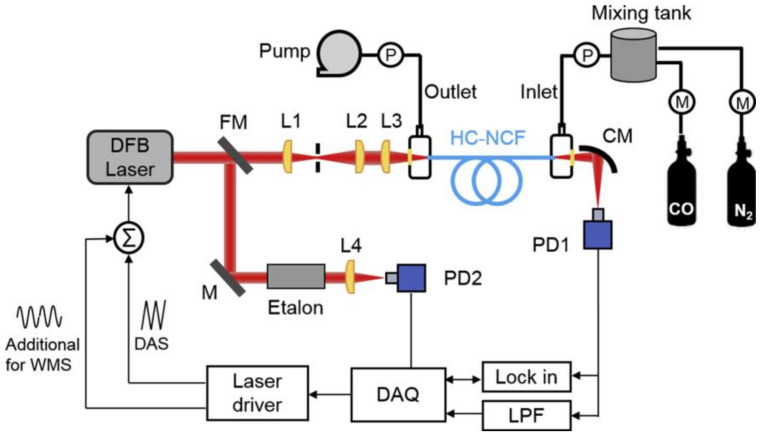
Experimental setup of the tunable diode laser absorption spectroscopy (TDLAS)-based CO sensor using the HC-ARF. FM: flip mirror; L: lens; M: mirror; CM: concave mirror; PD: photodetector; P: pressure gauge; M: mass flow meter; LPF: low-pass filter [95].

Yao et al. reported the sensitive carbon monoxide detection in a novel nodeless HC-ARF with TDLAS at the wavelength of 2.3 μm [95]. The HC-ARF consists of a single ring of eight nontouching silica capillaries around the air core, providing a single-mode light delivery of the 2.3-μm distributed feedback laser. The HC-ARF was used as a gas cell for the gas absorption measurement of a total path length of 85 cm. As the experimental setup shown in Figure 15, researchers implemented both DAS and WMS on the detection, and they demonstrated the detection limit of 0.4 ppm by detecting the CO line R (10) at 4297.7 cm^−1^ with the WMS technique. HC-ARFs that can deliver light in the MIR region with a low-mode noise can make ultrasensitive MIR gas sensing possible.

In 2018, Nikodem et al. demonstrated an in-fiber spectrometer in Kagome lattice HC-ARF for methane gas sensing in the MIR spectral region [65]. A 1.3-m-long HC-ARF that possesses a core diameter of 116 µm and low attenuation in the wavelength region around 3.4 µm was used as a gas cell, enabling efficient access to the strong fundamental transitions of methane around 3.33 µm. With an all-fiber differential frequency generation (DFG) source and the WMS technique, they developed a single ppm level resolution for methane detection. Later, they used the same Kagome lattice HC-ARF to perform methane detection in the MIR region [66]. Relying on the chirped laser dispersion spectroscopy technique, they achieved the lower methane detection limit of 500 ppb with the improvement of a factor of approximately 8.22 compared with the former report with the WMS technique [65]. The result shows the great potential of MIR gas sensors that use HC-ARFs and molecular dispersion spectroscopy.

**Figure 16:**
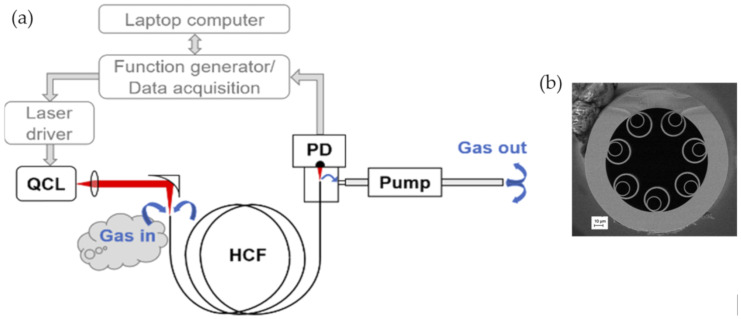
(**a**) Experimental setup of the nitrous oxide detection based on the laser absorption spectroscopy. QCL: quantum cascade laser; PD: photodetector; HCF: HC-ARF. (**b**) Cross-section of the HC-ARF with nested capillaries obtained with SEM [67].

In 2019, this group demonstrated the MIR laser absorption spectroscopy of nitrous oxide inside a silica-based revolver-type HC-ARF [67]. The emitting wavelength of the QCL is 4.53 µm, where the fiber enabled low-loss transmission, and then, the access to one of the strongest transitions of nitrous oxide could be efficient. As shown in Figure 16, this system demonstrated a detection limit at a single ppb level, using a 3.2-m-long fiber with a response time of less than 30 s.

In 2019, Yao et al. demonstrated the ultrasensitive MIR absorption spectroscopy of nitrous oxide by the use of a silica-based nodeless HC-ARF [94]. The core diameter of the fiber is 65 μm, and the inner cladding consists of a single ring of six nontouching circular capillaries. Based on the DAS, a distributed feedback ICL emitting at the wavelength of 3.6 μm was used to access the absorption line of nitrous oxide here for the high-resolution measurement. By optimizing the laser beam shape and using a low gas pressure, they suppressed the mode interference in the large hollow core and achieved a detection limit of 35 ppm for a fiber length of 120 cm, corresponding to a noise equivalent absorption coefficient at the level of 10^−7^ cm^−1^, which had never been achieved by the DAS performed with a large core HC-PCF.

In summary, the combination of HC-ARF with MIR laser-based spectroscopy has enabled sensitive gas detection in an extremely small sampling volume; thus, these proposed schemes can be further extended to other gases samples of interest. Moreover, with the development of the structure and material of HC-ARFs, MIR optics, and detection approaches, it is possible to realize compact, convenient, and high-quality trace gas sensing applications of HC-ARFs for both environment and human life.

## 7. Conclusions

Gas sensing plays an important role in our daily life and industry applications. Optical fiber gas sensors have huge demands due to their advantages of high sensitivity, outstanding anti-electromagnetic ability, and high reliability. In this review, we have given a brief description on the basic detection principles of the optical fiber gas sensors based on absorption spectroscopy, Raman spectroscopy, photothermal spectroscopy, and photoacoustic spectroscopy. Then, the development of optical fiber gas sensors in terms of fiber species, including MNFs, D-shaped fibers, and IG-PCFs is briefly reviewed. The performance of these sensors may be restricted due to the limited overlap of the evanescent field with gas. The focus of this paper is the use of HC-PCFs on gas sensing, including light-guiding mechanisms and properties of HC-PCFs, approaches to improving the gas-sensing performance, and recent research advances. The structural superiority of modified HC-PCFs enables fast gas diffusion and large light–gas overlap to obtain an enhanced sensing performance with short response time and high sensitivity. While flexible configuration and post-possessing of HC-PCFs allow gas in and out at the exposed end face or at the open side microchannel in the sensing elements, as well as in the remote and multiplexed multi-point gas detection system, and in the distributed gas sensing system. The larger hollow core, broader transmission band, and less mode coupling of the HC-ARF make it capable for MIR absorption-based gas sensing. Then, we summarize the recent advances on HC-PCF sensing and prospect their potential applications. For comparison, the recent published results for HC-PCF gas sensing performance were listed in Table A1 in the Appendix A, including the information of gas species, detection wavelength, sensing principle, type of fiber with its length, response time with assisted pressure, and detection limit. This review on the principle, configuration, approaches, and frontier research will promote a deep understanding of HC-PCF gas sensing and accelerate the development of advanced fiber gas sensors.

## Figures and Tables

**Table 1:** Absorption wavelengths and line strengths of 7 common gases in the near-infrared (NIR) region [18,34,101].

Gas	Absorption Line Wavelength (nm)	Line Strength (cm^−1^/molecule·cm^−2^)
Acetylene (C_2_H_2_)	1533	8.07 × 10^−21^
Ammonia (NH_3_)	1544	3.73 × 10^−22^
Carbon monoxide (CO)	1567	2.32 × 10^−23^
Carbon dioxide (CO_2_)	1573	1.94 × 10^−23^
Hydrogen sulfide (H_2_S)	1578	1.31 × 10^−22^
Methane (CH_4_)	1330	1.21 × 10^−22^
Methane (CH_4_)	1667	6.05 × 10^−22^
Water (H_2_O)	1365	2.12 × 10^−20^

## Appendix A

We have reviewed the recent published results for HC-PCF gas sensing performance. For comparison, we list the information of gas species, detection wavelength, sensing principle, type of fiber with its length, response time with assisted pressure, and detection limit in Table A1.

**Table A1:** Comparison of performance indicators for different HC-PCF gas sensors.

Gas	Wavelength	Sensing Principle	Type of Fiber/Length	Response or Averaging Time/Assisted Pressure	Detection Limit	Ref.
Acetylene(C_2_H_2_)	1510–1540 nm	DAS ^1^	HC-PBGF/ 5 m	240 s/ 0.7 bar	Not stated	[20]
	1528.03 nm	DAS	HC-PBGF/ 2 m	Sub minute/ <1 bar	<100 ppm	[61]
	1500–1550 nm	DAS	HC-PBGF/ 0.33 m	several minutes/ several bar	Not stated	[144]
	1530.1–1530.7 nm	WMS ^2^	HC-PBGF/ 13 m	Not stated	<1 ppm	[120]
	1530.371 nm	PTS ^3^	HC-PBGF/ 0.62 m	Not stated	2 ppb	[89]
	1510–1545 nm	DAS	suspended ring-core PCF/ 0.25 m	8.7 min/ 0.5 bar	Not stated	[63]
	1532.83 nm	WMS	HC-PBGF/ 0.094 m	Not stated	7 ppm	[84]
	1514–1538 nm	DAS	HC-PBGF/ 1 m	4 s/ 113 mbar	Not stated	[82]
	1518.71 nm	DAS	HC-PBGF/ 0.9 m	20 min/ 0.1 bar	500 ppm	[85]
Methane (CH_4_)	1315–1345 nm	DAS	HC-PBGF/ 1 m	4 s/ 113 mbar	Not stated	[82]
	~1666 nm	WMS	HC-PBGF/ 0.137 m	248 s/ not stated	158 ppm	[19]
	1665.48 nm	DAS	HC-PBGF/ 0.07 m	3 s/ 0.1 bar	647 ppm	[91]
	3.19–3.36 μm	FTS ^4^	HC-PBGF/ 0.8 m	2 min/ 2 bar	50 ppm	[83]
	3.33 μm	WMS	HC-ARF/ 1.3 m	<10 s/ 1.1 bar	Single ppm	[65]
	3.33 μm	CLDS ^5^	HC-ARF/ 1.3 m	1 s/ 1.1 bar	500 ppb	[66]
Ammonia (NH_3_)	1500 nm	DAS	HC-PBGF/ 1 m	4 s/ 113 mbar	Not stated	[82]
	1531.7 nm	DAS	HC-PBGF/ 1 m	Not stated	20 ppm	[88]
Nitrogen (N_2_)	584.5 nm	SRCS ^6^	HC-PBGF/ 1.5 m	36 s/ 6.9 bar	Not stated	[36]
	313–833 nm	RCS ^7^	HC-PBGF/ 0.3 m	10 s/ not stated	Not stated	[86]
Oxygen (O_2_)	559 nm	SRCS	HC-PBGF/ 1.5 m	36 s/ 6.9 bar	Not stated	[36]
	313–833 nm	RCS	HC-PBGF/ 0.3 m	10 s/ not stated	Not stated	[86]
Propane (C_3_H_8_)	535–545 nm 600–610 nm	SRCS	HC-PBGF/ 1.5 m	36 s/ 6.9 bar	Not stated	[36]
Ethane (C_2_H_6_)	535–545 nm 600–610 nm	SRCS	HC-PBGF/ 1.5 m	36 s/ 6.9 bar	Not stated	[36]
Carbon dioxide (CO_2_)	417–714 nm	RCS	HC-PBGF/ 0.3 m	3 min/ not stated	400 ppm	[86]
Toluene (C_7_H_8_)	313–1000 nm	RCS	HC-PBGF/ 0.3 m	10 s/ not stated	400 ppm	[86]
Acetone (C_3_H_6_O)	313–455 nm	RCS	HC-PBGF/ 0.3 m	10 s/ not stated	100 ppm	[86]
1,1,1-trichloroethane (1,1,1-C_2_H_3_Cl_3_)	313–455 nm	RCS	HC-PBGF/ 0.3 m	60 s/ not stated	12000 ppm	[86]
Hydrogen cyanide (HCN)	1530–1542 nm	DAS	HC-PBGF/ 1 m	4 s/ 113 mbar	Not stated	[82]
Hydrogen (H_2_)	1532.2 nm	SRGS ^8^	HC-PBGF/ 15 m	250 s/ 1.6 bar	17 ppm	[38]
Carbon monoxide (CO)	2.3 μm	WMS	HC-ARF/ 0.85 m	5 s/ 1.8 bar	0.4 ppm	[95]
Nitrous oxide (N_2_O)	3.6 μm	DAS	HC-ARF/ 1.2 m	Not stated	35 ppm	[94]
	4.53 μm	WMS	HC-ARF/ 3.2 m	23 s/ 0.94 bar	Single ppb	[67]

^1^ DAS: direct absorption spectroscopy; ^2^ WMS: wavelength modulation spectroscopy; ^3^ PTS: photothermal spectroscopy; ^4^ FTS: Fourier transform spectroscopy; ^5^ CLDS: chirped laser dispersion spectroscopy; ^6^ SRCS: spontaneous Raman scattering spectroscopy; ^7^ RCS: Raman scattering spectroscopy; ^8^ SRGS: stimulated Raman gain spectroscopy.
